# Book review: spectral domain optical coherence tomography in macular diseases

**DOI:** 10.1186/s40942-017-0072-0

**Published:** 2017-06-05

**Authors:** Müller Gonçalves Urias, Eduardo Büchele Rodrigues

**Affiliations:** Department of Ophthalmology, Federal University of São Paulo, R. Botucatu, 821, São Paulo, SP 04023-062 Brazil

**Keywords:** OCT, Optical coherence tomography, En face, Swept source, Spectral domain

## Abstract

This is a review of the book Spectral Domain Optical Coherence Tomography in Macular Diseases edited by Meyer et al. (Spectral domain optical coherence tomography in macular diseases. Springer, New York, [2017]). The book provides clinicians both basic and advanced understanding of OCT applications in the field of medical retina.

## Background

The outstanding book Spectral Domain Optical Coherence Tomography in Macular Diseases edited by Carsten et al. [1]. Optical coherence tomography (OCT) is the optical analog of ultrasound examination and employs a technique called low-coherence interferometry to obtain high-quality cross-sectional and en face image. OCT allows non-invasive, high resolution, in vivo evaluation of biological tissues. OCT is the most important imaging test in ophthalmology and is currently used both in the diagnosis and management of most vitreo-retinal disorders.

## Main text

The book Spectral Domain Optical Coherence Tomography in Macular Diseases was produced by three brilliant Editors and over 60 Chapters authors. This 1st Edition by Springer Publisher. By gathering some of the most famous experts in the field to contribute chapters, Profs. Meyer, Saxena and Sadda were able to offer a superb work with several key features including point-to-point correlation of retinal conditions and spectral-domain OCT, 3-dimentional evaluation and segmentation analysis and the surgical use of OCT in vitreoretinal diseases management. The result is a streamlined, efficient teaching book that should be enjoyed as reference text for clinical practice in retina.

The book covers OCT in detail and comprehensively in five parts throughout the more the 400 pages. In the first part, the readers may appreciate a detailed review of all technologies available on OCT including en-face, OCT-angiography, and swept-source OCT. Worthy to highlight is the excellent introductory chapter by Sisha et al. named OCT: a Primer. In the second chapter, the role of OCT for the most common retinal diseases are presented. The OCT images are of high quality and well labeled. The focus was not only the diagnostic importance of OCT test, but also its importance in clinical decision making. The third part reviews the use of OCT in diseases caused by anomalous vitreous adherence, whereas the fourth covers few interesting OCT investigations in not so common but also important retinal conditions. Finally, the last fifth part summarized and provided future directions to the revolutionary OCT technology.

## Conclusions

The new book Spectral Domain Optical Coherence Tomography in Macular Diseases provided clinicians both basic and advanced understanding of OCT applications in the medical field. The book will be useful for residents and fellows interested in pursuing an education in the retina medical specialty as well as practicing ophthalmologists. Overall, it has included the multiple applications and interpretation of OCT related to the posterior segment of the eye. Advances in OCT technology provided better understanding of pathogenesis, enhanced monitoring of progression and evaluation of response to therapy in diseases of the retina. Further improvements in either software or hardware should further advance the clinician’s ability to assess and manage chorioretinal diseases. We strongly suggest the book to all colleagues interested in res clinical applications of OCT in ophthalmology and retina.

## Abbreviation

OCT: optical coherence tomography
